# Microfluidic generation of nanoparticles using standing wave induced ultrasonic spray drying†

**DOI:** 10.1039/d4na01012d

**Published:** 2025-03-06

**Authors:** Holger Bolze, Keiran Mc Carogher, Simon Kuhn

**Affiliations:** a KU Leuven, Department of Chemical Engineering Celestijnenlaan 200F 3001 Leuven Belgium simon.kuhn@kuleuven.be; b Institut für Medizintechnik, Otto von Guericke Universität Magdeburg Universitätsplatz 2 39106 Magdeburg Germany

## Abstract

Spray drying is a well-established process for generating particles for various applications, including pharmaceuticals. In this process, atomization plays a crucial role by defining the size of the droplets and, consequently, particle size. While ultrasound is commonly used to enhance atomization by reducing droplet size, a novel approach has been introduced that utilizes plug flow to generate plugs resonating with an applied ultrasound frequency, triggering surface atomization. This study investigates the applicability of this method for microfluidic atomization and spray drying, particular for pharmaceutical carrier particles. The generated droplets exhibit a size of 7.24 μm and a PDI of 0.18, indicating a monodisperse distribution. The droplets are produced in discrete burst events, enabling an energy-efficient pulsed process with an applied power of less than 1 W. This approach successfully generates lipid nanoparticles with an average size of 140 nm, underscoring its potential for nanoparticle production.

## Abstract 2

This study introduces an ultrasonic spray drying method that uses acoustic resonance for energy-efficient atomization, producing μm-sized droplets and pharmaceutical nanoparticles with precise size control.

## Graphical abstract

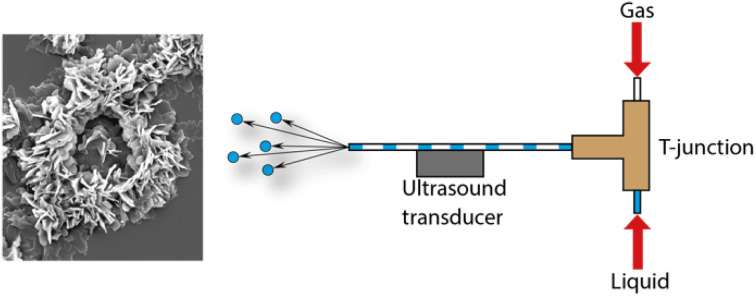

## 1 Introduction

For many applications, the formulation of substances is important to change their quality.^1–7^ By formulating a substance as a particle or droplet, its applicability can be significantly influenced by increasing the surface area of the substance, improving dosability and creating the possibility of functionalising the phase boundary.^1,4,8^ There are different processes to produce solid particles, either by compartmentalising a bulk material (*e.g.* grinding, emulsification) or by assembling a particle from smaller units (*e.g.* polymerisation, crystallisation).^8^ A common industrial process for formulating solid substances into particles smaller than a few millimetres is spray drying.^9^ For this, the substances to be formulated must be dissolved in a liquid which is atomized into small droplets in the gas phase. The solvent starts to evaporate and the substance starts to form a solid particle. Due to compartmentalisation, the growth of the particle is restricted to the material present in the droplet.^10^ This process requires two important steps. Firstly, the atomization device which distributes the liquid, and secondly the drying chamber, which must create the conditions for solvent evaporation, removal of the solvent vapours, and prevention of particle agglomeration before they are completely dry.^10^

Since droplet size and variance are determined by atomization, a good dispersion process is one of the keys to produce small and monodisperse particles.^9,11,12^ For this reason, various dispersion techniques have been developed and are used in research and industry.^3,10^ The most common macroscopic techniques involve nozzles whose small diameter leads to atomization upon leaving the pressurized nozzle.^3,10^ To reduce droplet size, the process can be enhanced, for example, by a colliding air stream or sonication to promote the breakage of individual droplets.^3,10,13^ More recently, microfluidic channels are used to further reduce the droplet size at the expense of limited throughput.^14–16^

Ultrasound, on the other hand, has been widely integrated into microfluidic systems for various applications.^17^ It is commonly used to enhance fluid mixing^18–20^ or to mitigate fouling in microfluidic channels.^21–23^ Other applications include particle focusing in fluid flow,^24–26^ emulsification,^27–29^ and radical generation through cavitation.^30^ Ultrasound has also been employed for liquid atomization; however, to our knowledge, only surface acoustic waves (SAWs) have been utilized to generate droplets outside the confines of microfluidic channels.^31–33^ An important distinction between these applications lies in the ultrasound frequency applied. Depending on the frequency, different acoustic phenomena can be induced, enhancing the versatility of the process.^34–36^ Achieving acoustic resonance, which is crucial for effective processing, requires that the ultrasound frequency aligns with the size of the feature being affected.^17^

In our research, we analyzed the use of ultrasound to trigger acoustic atomization through acoustic resonance along the length of liquid plugs, whose lengths can be easily adjusted to match the desired operating frequency. The generated droplets can be dried into nanoparticles with a monodisperse size distribution. Atomization based on ultrasound resonance is energy efficient, easily scaled up, and requires only a drying chamber of limited size.

## 2 Materials and methods

Two experimental setups were employed: (1) an atomization analysis setup to investigate droplet generation parameters and (2) a particle harvest demonstrator to assess nanoparticle formation.

### 2.1 Atomization analysis setup

**Fig. 1:**
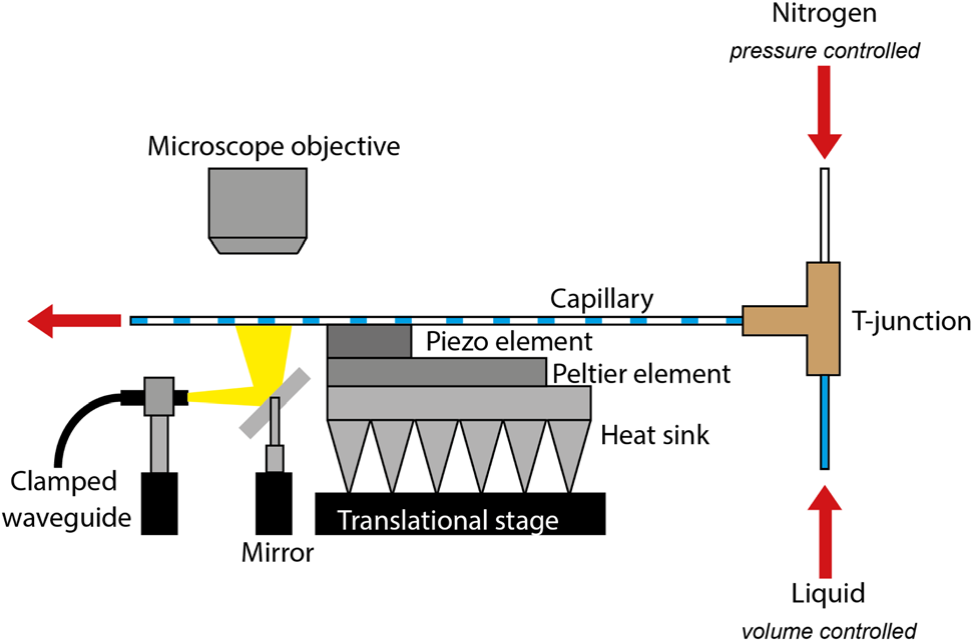
Schematic of the atomization analysis setup.

A schematic of the atomization setup is shown in Fig. 1. The system consists of a 1 mm inner diameter (100 mm in length, 1.2 mm outer diameter) square glass capillary (CM Scientific Ryefield (EU), Dublin, Ireland) affixed to a 32 mm × 32 mm × 4 mm piezoelectric element (C-205, Fuji Ceramics Corporation, Fujinomiya, Japan) using an epoxy-based adhesive (PT37, Pacer Technology, Rancho Cucamonga, CA). The setup is mounted on a Peltier element (TES1-12704, RS Components, Corby, United Kingdom) with a heat sink and fan (RS PRO 703-3451, RS Components, Corby, United Kingdom) for thermal regulation. A signal generator (DG1032, Rigol Technologies, Portland, OR) and amplifier (1040L, Electronics & innovation, Rochester, NY) drive the piezoelectric element at its resonance frequency, determined *via* impedance analysis (model 16777k, SinePhase Instruments, Hinterbruehl, Austria).

Plug flow is generated *via* a T-junction (P-713, IDEX Health & Science, Rochester, NY) using PTFE tubing (0.5 mm inner and 1.2 mm outer diameter, IDEX Health & Science, Rochester, NY). To generate plug flow, the T-junction was connected to two vials: one empty and the other containing the liquid. Both vials were pressurized using nitrogen, controlled by a pressure controller with an integrated mass flow controller (OB1 with MFS-D-3, Elvesys, Paris, France), utilizing the same tubing for both. The outlet of the capillary was connected to another tubing, which collects the liquid in a small beaker. The entire setup was placed on a precision translation stage (XYT1/M, Thorlabs, Newton, NJ), and a mirror (PF1011-P01, Thorlabs, Newton, NJ) was used to illuminate the capillary from below using an external light source (KL 2500, Schott, Mainz, Germany). The capillary was placed below a microscope (SMZ25, Nikon, Tokyo, Japan) equipped with a high-speed camera (Fastcam mini UX100, Photron, Tokyo Japan) to capture the atomization events.

Preliminary studies were conducted using water dyed with methylene blue (1 mg mL^−1^, Sigma-Aldrich, St. Louis, MO) and 7 W of applied ultrasonic power. For standardized experiments using acetone as solvent, the capillary was sonicated at its resonance frequency of 470 kHz and 3 W of applied power. For pulsed sonication, a 2 ms ultrasound pulse followed by 20 ms of relaxation time was implemented, together with an increased peak power to establish an average applied ultrasound power of 1 W. If not stated otherwise, acetone was pumped at a rate of 84 μL min^−1^ through the system, while nitrogen was injected with 150 mbar to generate plug flow. To enhance the contrast, the acetone contained 1 mg per mL malachite green (Sigma-Aldrich, St. Louis, MO). For experiments analyzing the effect of changing interfacial tension, Tween 20 (9.86 mg mL^−1^) or Tween 80 (10.64 mg mL^−1^) was added (Sigma-Aldrich, St. Louis, MO).

**Fig. 2:**
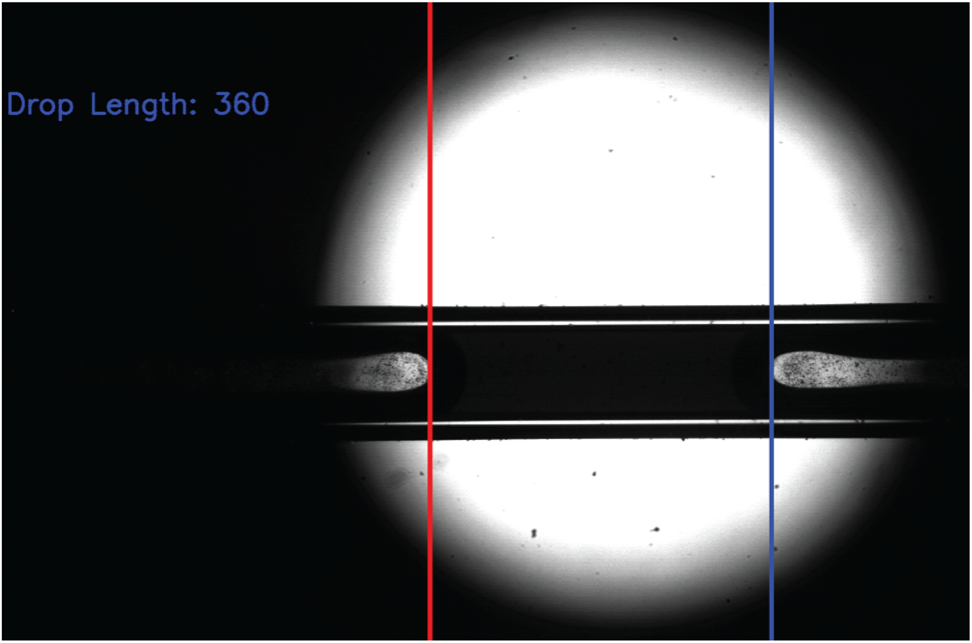
Example image of plug flow in the capillary and the automatic extraction of the plug length. Included is the size of the plug (named drop in the program) in pixels as measured by the program.

The atomization events were analyzed in terms of length of the liquid plugs and if they generate liquid droplets. A change in greyscale value in the images was used to detect the phase boundaries and to extract the plug length (see Fig. 2 for an example image). The plug was tracked on subsequent images to calculate its average size. The atomized droplets themselves were analyzed in terms of their size (using the analyze particles function in ImageJ^37^) and velocity.

### 2.2 Particle harvest demonstrator

The above described setup was capable to analyze the droplet generation, but was not suitable to visualize, analyze or harvest generated nanoparticles due to a lack of resolution and the resuspension of generated nanoparticles. To demonstrate the capability of the proposed setup to generate nanoparticles, the analysis setup was modified to harvest nanoparticles. Therefore, the tubing connected to the outlet of the capillary was replaced by a 3D-printed protection (High temperature resin, Formlabs, Somerville, MA) to prevent liquid back flow onto the piezoelectric element. The liquid dripping from the capillary was collected in a small beaker, while the released atomized droplets impact on a target placed 2 cm from the capillary outlet. The targets consisted either of paper (spray pattern visualization) or glass (nanoparticle harvesting).

The liquid was acetone containing either 0.228 mg per mL methylene blue for atomization quantification or 0.7 mg per mL trimyristine and 1 mg per mL polysorbate 80 for particle generation (Sigma-Aldrich, St. Louis, MO). The liquid was pumped at a flow rate of 84 μL, while the gas phase was delivered at 150 mbar overpressure to generate a gas dominated plug flow. After screening for optimized droplet production, the piezoelectric element was excited at its resonance frequency of 356.8 kHz in pulsed mode (2 ms ultrasound pulse followed by 20 ms of relaxation) resulting in an average applied power of less than 1 W for atomization. The generated nanoparticles were collected from the target and analyzed using DLS (120°, 3D-DLS capable custom-made setup, LS Instruments, Fribourg, Switzerland) or SEM (JSM-6010LV, JEOL Ltd, Tokyo, Japan).

## 3 Results

### 3.1 Atomization analysis setup

The experimental setup was designed to determine the optimal plug size required for efficient atomization and to assess the size distribution of the generated droplets.

#### 3.1.1 Suitability of microfluidic atomization for droplet generation

While the general effect of atomization has been previously described by Mc Carogher *et al.*,^38^ it is crucial to analyze this effect within a setup specifically designed for droplet characterization, in order to interpret the results and assess the viability of droplet generation for this system. To replicate the atomization effect, water was chosen as the working fluid. Initially, the influence of the adjusted plug length on atomization was evaluated, since, according to the aforementioned study, plug length is a key parameter in initiating the atomization phenomenon.

For this analysis, images were captured 15 mm downstream of the piezoelectric element, using an illumination spot size of approximately 5 mm. Under silent conditions (15 μL min^−1^ flow rate of water and 70 mbar gas pressure), the mean plug length was found to be 2.37 mm with a standard deviation of 0.08 mm. Upon the application of ultrasound (7 W at 476.2 kHz), the average plug length decreased by 13%, with the standard deviation increasing to 0.16 mm. This variation in plug size can be attributed to the forces exerted by the ultrasound, which apply pressure on the plugs depending on its distance to the pressure nodes. Consequently, this results in a directed flow of water between plugs through the wall film, altering the plug length depending on both the location within the channel and the residence time.

**Fig. 3:**
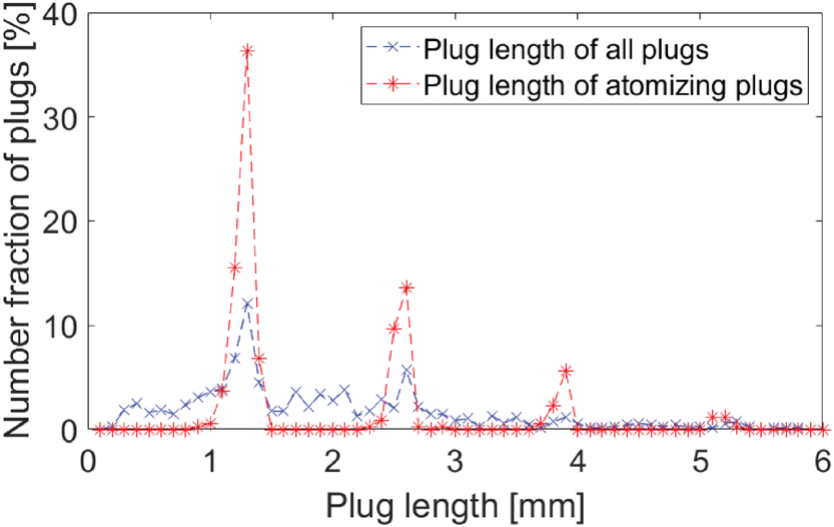
Comparison of the full plug population with the subset of atomizing plugs in an experiment conducted under sonicated conditions. The data represents a singular measurement using the described conditions for aqueous experiments.

As indicated in the preliminary study, only specific plug lengths induce atomization (see Fig. 3). In the current experimental setup, atomization was observed for plug lengths of 1.3 mm (and its multiples), with no atomization occurring for plugs deviating more than 200 μm from these values. The atomization effect is contingent upon the formation of a standing wave within the liquid plugs, as demonstrated in previous studies.^38,39^ Thus, only plugs that conform to the resonance frequency (or its multiples) will initiate the atomization process. Moreover, the data presented in Fig. 3 suggests that when considering the entire plug population, plugs that match the resonant length not only undergo atomization but also appear more frequently than plugs of non-resonant sizes. This indicates that the ultrasound modulates the flow profile, thereby enhancing the occurrence of plugs that align with the resonant frequency.

To optimize the volume of liquid atomized, it is essential that the plugs exhibit a precise length, with deviations of less than 0.2 mm. This requirement necessitates a high degree of precision in plug length control. However, the changes in plug size induced by sonication complicate such precision control. On the other hand, the focusing effect of ultrasound on the required plug length makes the process feasible without stringent control of plug size, which simplifies the design and construction of the system.

#### 3.1.2 Temporal structure of atomization events

High-speed camera footage was analyzed to characterize the temporal dynamics of atomization events. The measurements were conducted under the same operational parameters as previously described (15 μL min^−1^ water flow, 70 mbar gas pressure, 7 W, and 476.2 kHz ultrasound frequency). Image analysis revealed that ultrasound resonance did not result in a continuous generation of atomized droplets. Instead, atomization occurred in discrete bursts, each releasing a varying number of droplets.

**Fig. 4:**
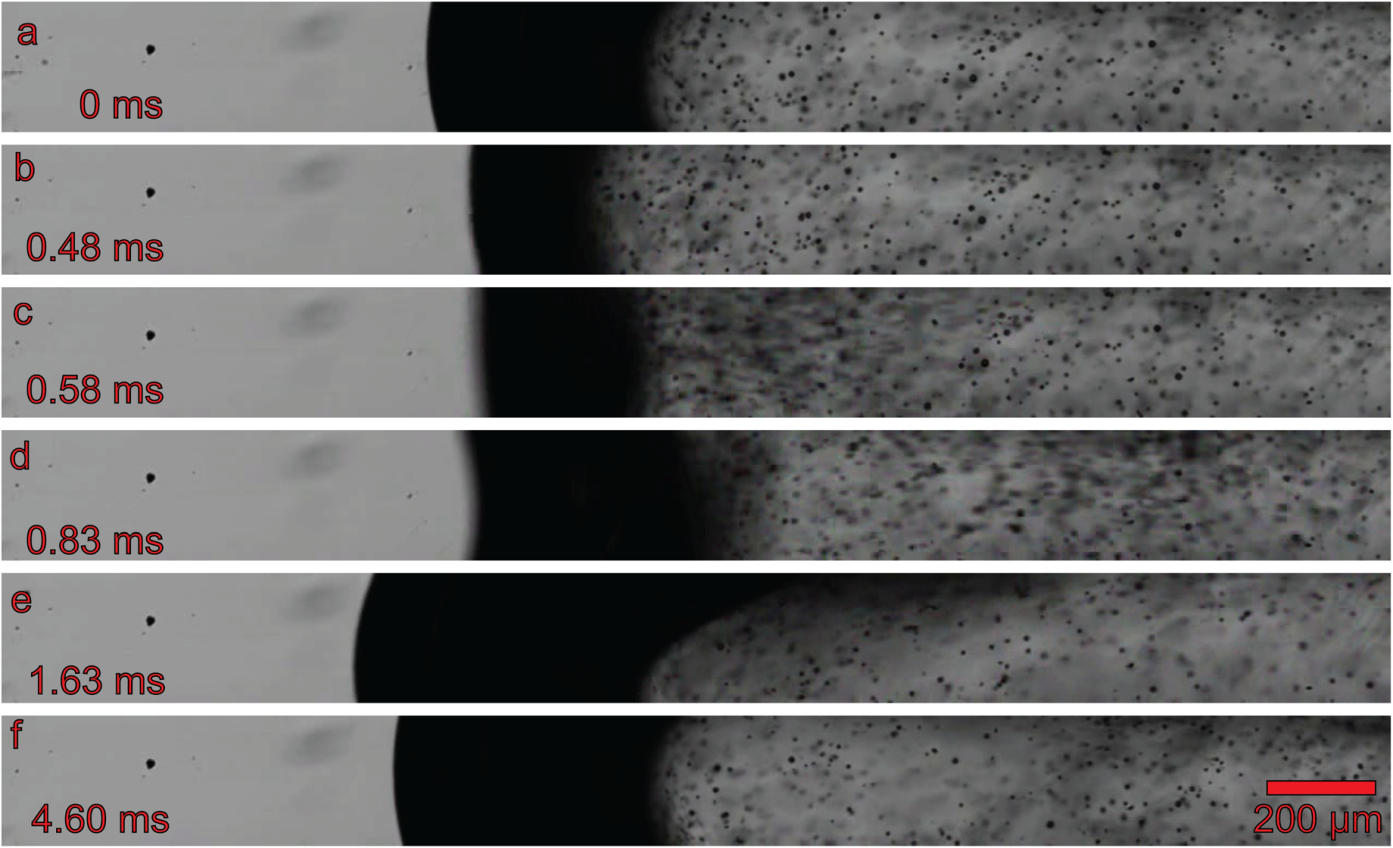
High speed imaging of an ultrasound induced atomization event. For this analysis, the capillary was recorded at 40 000 frames per second and a shutter rate of 100 000 frames per second. The images represent a time series of the burst process: (a) initial state; (b) before the burst; (c) high speed ejection of particles; (d) slowing down of particles; (e) disrupted interface; (f) interface after the burst.

Prior to sonication, the liquid formed a concave surface due to the hydrophilic capillary material (Fig. 4a). Just before the onset of atomization (at 0.58 ms), the gas–liquid interface flattened (Fig. 4b). Following the initiation of atomization, droplets were ejected from the surface at such a high velocity that they could not be resolved as individual particles (Fig. 4c and d), with ejection speeds exceeding 2.4 m s^−1^. These droplets decelerated rapidly due to the absence of sustained propulsion, combined with their relatively low mass (Fig. 4e). As a result, the droplets were slowed to below 0.2 m s^−1^ within 1.5 ms.

During the atomization event, the gas–liquid interface exhibited vigorous movement. In the intervals between atomization bursts, the interface returned to its equilibrium shape (Fig. 4f). The temporal interval between atomization events, at an applied frequency of 470 kHz, was measured to be 14.84 ms, with a standard deviation of 5.53 ms. This temporal pattern can be attributed to the loss of acoustic resonance as the gas–liquid interface is distorted, leading to the collapse of the standing wave in the liquid plug. Once the interface returns to its equilibrium position, the standing wave is re-established, facilitating subsequent atomization events.

The droplets initially remain suspended in the gas phase; however, over time, they begin to coalesce with neighboring droplets and merge with the gas–liquid interface. As the capillary walls are coated with a thin liquid film, all droplets (or dried particles) eventually re-enter the solvent phase. The overall lifetime of the droplets is limited to approximately 1.03 s, underscoring the importance of their rapid removal from the capillary for any subsequent applications.

#### 3.1.3 Adapting the system for particle generation

While water is a widely used solvent and safe in case of atomization and vaporization, it's comparatively high vapor pressure and surface tension are detrimental for its usage in microfluidic atomization. Therefore, acetone was used as a solvent instead for nanoparticle generation, also to generate solid carrier particles stable in water.

Using the same setup, a similar behavior as for water was measured including atomization, change of plug size, and the burst structure of its generation. The major difference upon switching the solvent was that the applied ultrasound energy had to be reduced. This was most probably an effect of the decreased interfacial tension of acetone (24.5 mN m^−1^ compared to 72 mN m^−1^ of water^40^), which makes it easier to disturb the interface and to create droplets. As a result, 3 W of applied ultrasound power was chosen for experiments using acetone to yield sufficient atomization efficiency.

#### 3.1.4 Pulsed ultrasound

**Fig. 5:**
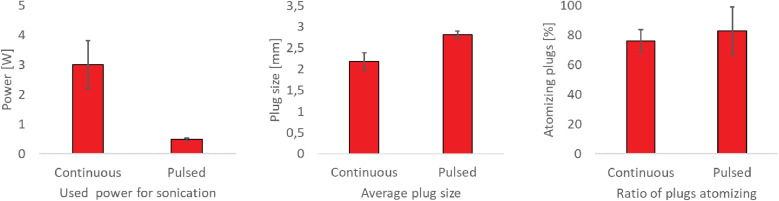
Comparison of continuous and pulsed sonication.

As outlined above, constant sonication does not result in constant atomization events. A pulsed ultrasound mode (2 ms activation, 20 ms relaxation) reduces average power consumption below 1 W (Fig. 5), minimizing thermal effects and simplifying system cooling requirements, while generating a comparable number of atomizing plugs. This mode proves advantageous for energy-efficient spray drying processes and all further experiments were performed in pulsed mode.

#### 3.1.5 Droplet size

**Fig. 6:**
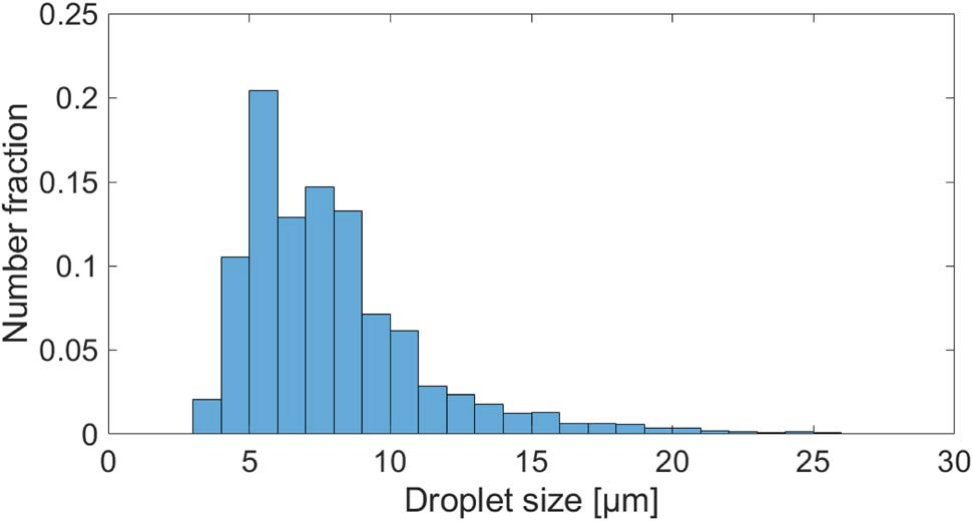
Histogram of droplets generated at standard conditions in multiple experiments. Based on more than 7000 measured droplets.

Droplets generated in acetone exhibit an average size of 7.24 μm with a PDI of 0.18, indicating a monodisperse distribution. This size is, according to literature, not the smallest size reported for spray drying apparatus,^41,42^ but at the lower end for spray drying processes and smaller than most industrially used designs.^43–45^ The droplet size distribution is depicted as a histogram in Fig. 6, from which a tailing towards larger droplet sizes is evident, indicating the aforementioned droplet coalescence.

Usual spray drying processes aim at tuning the particle size to the application, therefore we applied a surfactant to show the feasibility of changing the droplet size in a controlled manner. The results are tabulated in Table 1, and the addition of surfactant was able to reduce the droplet size by 16%, which corresponds to a reduction in droplet volume of 40%. In comparison, Tween 20 seem to have higher impact than Tween 80 on the atomization (5% smaller droplets) when using the same molar concentration. Hence, it is possible to adjust the atomized droplet size by changing the interfacial tension with surfactants.

**Table 1:** Impact factors and the resulting parameters

Parameter	Size [μm]	Standard deviation [μm]	PDI
None	7.24	3.31	0.18
Tween 20 (0.99 mg mL^−1^)	6.65	3.37	0.20
Tween 80 (0.7 mg mL^−1^)	7.01	2.84	0.14
Tween 80 (1.4 mg mL^−1^)	6.10	2.20	0.11

### 3.2 Particle harvest demonstrator

**Fig. 7:**
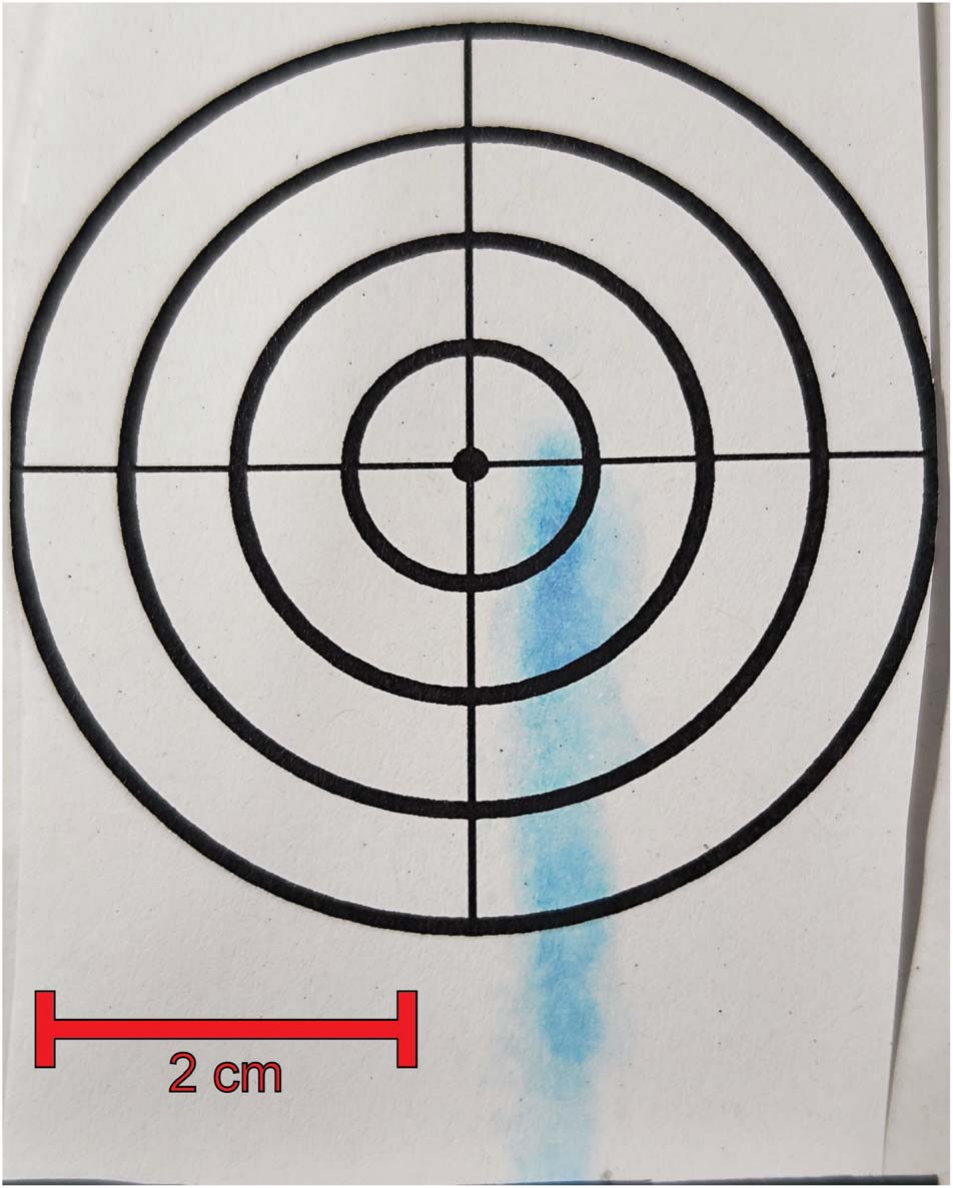
Spray pattern visualized using a paper in the containment chamber.

The primary objective of this experimental setup was to demonstrate the feasibility of utilizing the atomization process for generating particles suitable for use as pharmaceutical carriers. To visualize the impact of the droplets and the resulting spray pattern, a paper target was positioned on the rear wall of the containment chamber (Fig. 7). The analysis revealed that the majority of droplets impacted a small area directly in front of the capillary. The resulting spray pattern exhibited a width of 4 mm and a height of 25 mm. Consequently, it is evident that focusing on the region in front of the capillary is sufficient for efficiently collecting the generated particles.

**Fig. 8:**
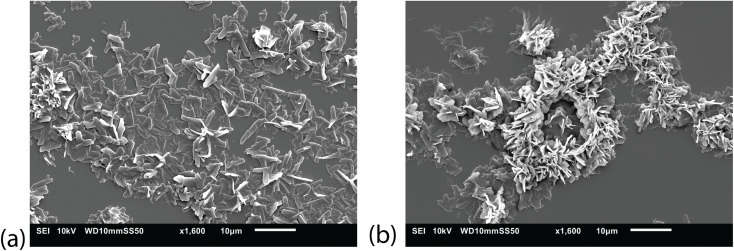
SEM pictures of dried trimyristine particles. (a) showing singular platelets, while (b) shows a tight agglomerate of platelets.

Subsequently, the particle generation characteristics of the setup were investigated using trimyristine, a lipid commonly used in pharmaceutical applications. The lipid solution was sprayed onto a glass slide to capture the particles, which were then analyzed using SEM. The SEM analysis confirmed the presence of particles on the glass surface (Fig. 8). The particles exhibited a plate-like morphology, which is typical of dried trimyristine particles. Although some individual plates were observed, the majority of the particles formed a dense layer composed of variously oriented plates. A plausible explanation for the observed particle structures is the incomplete drying of particles, which causes them to merge upon impact, while keeping their angle of impact.

For size distribution analysis, DLS was employed. The particle sample was suspended in water and subjected to sonication in a cleaning bath. This bath had sufficient energy to break apart any agglomerates formed during storage, yet was too weak to disintegrate the solid particles. The DLS analysis yielded a measured hydraulic diameter of 140 nm, which is below the 200 nm cutoff threshold necessary for carrier particles to enter cells. However, the polydispersity index (PDI) of the sample was 0.79, indicating highly polydisperse particle size distribution. This wide distribution is most likely attributed to the suboptimal drying process employed during particle generation.

## 4 Discussion

The observation that atomization is triggered only every 15 ms suggests that the system operates in a manner where a significant portion of the sonicated time is spent without particle production. Nevertheless, this pulsed mode enables the delivery of acoustic energy precisely when required, thereby enhancing the overall efficiency of the atomization process. The generation of small, monodisperse droplets with an average size of 7.24 μm and a generated particle size of 140 nm highlights the potential of this technique for producing pharmaceutical carrier particles. Furthermore, the ability to modulate droplet size by adjusting interfacial tension provides an additional degree of control, achievable through the use of surfactants. However, this control introduces additional formulation considerations to ensure the desired surface tension is achieved.

The particle harvest demonstrator successfully fulfilled its primary objective of producing nanoparticles suitable for pharmaceutical applications from resonating liquid plugs. The observed agglomeration and the elevated polydispersity index of the particles indicate that there is potential for optimizing the drying process. Nevertheless, the particle size distribution is already well within the acceptable range for a demonstrator, reinforcing the system's potential for further refinement and development.

## 5 Conclusions

This study demonstrates a novel ultrasonic spray drying method that generates sub-10 μm droplets and pharmaceutical nanoparticles. The process leverages acoustic resonance for energy-efficient atomization and allows for precise control over droplet size. These findings provide a foundation for developing scalable microfluidic-based nanoparticle synthesis for biomedical applications.

## Data availability

The data supporting this article have been included as part of the ESI.†

## Author contributions

H. B.: conceptualization, methodology, investigation, writing – original draft; K. M. C.: methodology, writing – review & editing; S. K.: writing – review & editing, supervision, funding acquisition.

## Conflicts of interest

There are no conflicts to declare.

## Supplementary Material

NA-007-D4NA01012D-s001

NA-007-D4NA01012D-s002

NA-007-D4NA01012D-s003

NA-007-D4NA01012D-s004
